# Application of different aquatic plants in an alternated fill and drain wetland system of Phetchaburi municipal wastewater treatment in Thailand

**DOI:** 10.1007/s11356-023-31266-1

**Published:** 2023-12-01

**Authors:** Onanong Phewnil, Kasem Chunkao, Paiboon Prabhuddham, Thanit Pattamapitoon

**Affiliations:** 1 Department of Environmental Science, Faculty of Environment, Kasetsart University, Bangkok, 10900 Thailand; 2 The King’s Royally Initiated Laem Phak Bia Environmental Research and Development (LERD) Project, The Chaipattana Foundation, Ban Laem District, Phetchaburi Province 76100 Thailand

**Keywords:** Aquatic plant, Municipal wastewater, Wetland, Alternate flooding and drying, *Canna* and *Heliconia*

## Abstract

This study evaluated the treatment efficiency of municipal wastewater from Phetchaburi in Thailand in an alternated 5-day flooding and 2-day drying wetland system with two plants species, namely, *Canna indica* and *Heliconia psittacorum*. The efficiencies of biochemical oxygen demand (BOD5) treatment were in the ranges of 90.5% ± 4.8% and 86.9% ± 7.3% for *Canna* and *Heliconia*, respectively. Those of chemical oxygen demand (COD) treatment were in the ranges of 75.5% ± 7.9% and 75.3% ± 9.0% for *Canna* and *Heliconia*, respectively. Both plants’ removal efficiencies of TN, NH4-N, and TP were greater than 40%. Lead and cadmium accumulation in both plants significantly differed between the upper and lower parts of the plants. However, the lead and cadmium accumulation in *Heliconia* were greater than their accumulation in *Canna.* Although *Canna* had a higher nutrient removal efficiency than Heliconia, there are many varieties of *Canna* in Thailand. These results indicate that the variety of *Canna* does not affect the nutrient removal efficiency. In conclusion, a wetland system with alternated flooding and drying conditions can be applied in communities where BOD_5_ and COD are the dominant wastewater pollution characteristics. Both ornamental plants are suitable absorbents for lead and cadmium, and although the accumulation is lower in *Canna* than in *Heliconia* for both heavy metals, the difference was not significant.

## Introduction

Constructed wetlands are employed worldwide for household and industrial wastewater treatment because of their low cost, ease of operation, and low maintenance. Generally, household wastewater is contaminated with organic substances. The pollutants in municipal wastewater (MW) can be mainly characterized by the biochemical oxygen demand (BOD), chemical oxygen demand (COD), total nitrogen (TN), ammonium nitrogen (NH_4_-N), and total phosphorus (TP) (Chunkao et al. 2012; Phewnil et al. 2014). Phetchaburi Municipality is in Phetchaburi Province, Thailand, around 150 km from Bangkok, Thailand. The municipality has a population of approximately 44,000 persons and produces around 6000–8000 m^3^ of wastewater per day. In the past, wastewater from Phetchaburi Municipality was drained into Phetchaburi River without any treatment.

Consequently, the water quality in the river deteriorated. The King’s Royally Initiated Laem Phak Bia Research and Development Project (LERD project) was established in 1990. The project site is located in Ban Laem District in Phetchaburi Province. Phetchaburi MW was conveyed through an 18.5-km-long underground high-density polyethylene pipe from the town to the project for treatment. The wastewater treatment system of the LERD project consists of four systems: an oxidation pond, a grass filtration system, constructed wetlands, and a mangrove forest filtration system. Most developing countries are trying to adapt various aquatic plants for wastewater treatment systems, even though the body of knowledge about applying aquatic plants in wetlands wastewater treatment systems is incomplete because of plant, soil, climatic conditions, and pollutant characteristics (Zhang et al. 2009; Jampeetong et al. 2012). Wetlands play a key role in removing nutrients or pollutants *d* in wastewater via biological processes that depend on the plant species used, the hydraulic loading rate, biomass, microbial activity, and soil (Calheiros et al. 2007; Stefanakis et al. 2014). The most important functions of aquatic plants in constructed wetlands are related to their physical characteristics, such as the root type, growth rate, nutrient uptake, heavy metal accumulation, microbial activity in the root zone, and oxygen transfer in the systems. Vertical flow constructed wetlands (VFCWs) with alternating 5-day flooding and 2-day drying is an alternative for wetland wastewater treatment because microbial activities will exudate enzymes specific for substrates. Organic nitrogen will be transformed to ammonium and nitrate by microbial enzymes in the rhizosphere (Kong et al. 2009; Zhang et al. 2010). VFCW systems are intermittently flooded and drained, allowing air to refill the substrate pores within the bed. Nitrification occurs during drying, and denitrification occurs under flooding (Edwards et al. 2006; Li et al. 2013). Therefore, alternated flooding and drying are highly effective for removing the nutrients in VFCW.

*Canna* and *Heliconia* are ornamental emergent plants used in the wetlands to increase the aesthetic and economical values the treatment site (Konnerup et al. 2009; Abou-Elela and Hellal 2012; Khan et al. 2021). Canna and Heliconia are ornamental emergent plants used in the wetlands to increase the aesthetic and economical values the treatment site. Canna and Heliconia were employed for environmental beautification and to encourage public acceptance of wastewater treatment systems, which demonstrated great efficiency in treating total suspended solids (TSS) by more than 92%, chemical oxygen demand (COD) by 88%, and biochemical oxygen demand (BOD) by 90%. Furthermore, they exhibited the capability to effectively removing heavy metals. Canna exhibited a faster growth rate than Heliconia, but the latter was used for ornamental purposes, thus contributing to economic value addition. Additionally, these plant species were utilized for small-scale community wastewater treatment and in rural areas of various countries, including China, Iran, India, Oman, Pakistan, and Turkey (Konnerup et al. 2009; Abou-Elela and Hellal 2012; Khan et al. 2021).

In the broad application of constructed wetlands for wastewater treatment, numerous studies have proposed various flow methods, categorizing them as hybrid-constructed wetlands (HCW), vertical flow constructed wetlands (VFCW), and horizontal flow constructed wetlands (HFCW). However, many studies remain inconclusive due to the wide range of hydraulic retention times in the flow mechanisms, which can affect the nitrification and denitrification rates of the treatment system, was well as no clear pattern was suggested in the pollutant removal efficiency of ornamental plants (García-Ávila et al. 2023). The purpose of this study was to investigate the growth rate, nutrient removal efficiency, and heavy metal accumulation in *Canna* and *Heliconia* when applied in a VFCW system and its applicability to tropical areas. In while the study is divided into two parts as it compares the treatment between the two species (*Canna* and *Heliconia)* and in later part examines on three species of *Canna* spp. to determine their suitability based on their physiology and practicality for the treatment of municipal wastewater.

## Methodology

### Site description

**Fig. 1:**
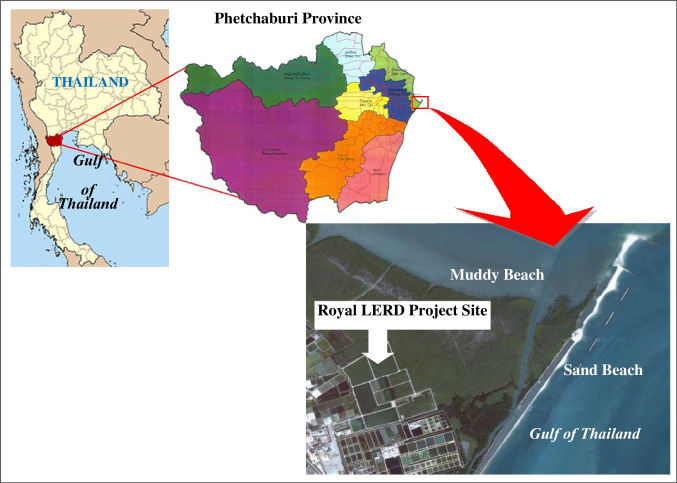
Location of King’s Royally Initiated Laem Phak Bia Research and Development Project (LERD Project), Phetchaburi Province, Thailand

The experimental facility used in this study is in the King’s Royally Initiated Laem Phak Bia environmental Research and Development project (LERD), 160 km from Bangkok 160 km in Laem Phak Bia Sub-District, Ban Laem District, Phetchaburi Province, Thailand. The site is about 18 km away from Phetchaburi Municipality. The project site is behind the second growth of a mangrove forest that extends along a mud beach adjacent to the Gulf of Thailand, as shown in Fig. 1. The LERD project aims to study and conduct research and development activities on community garbage disposal and wastewater treatment under the King’s initiation. For this purpose, the project adopts natural processes, simple technology, and local materials.

### Experimental units

**Fig. 2:**
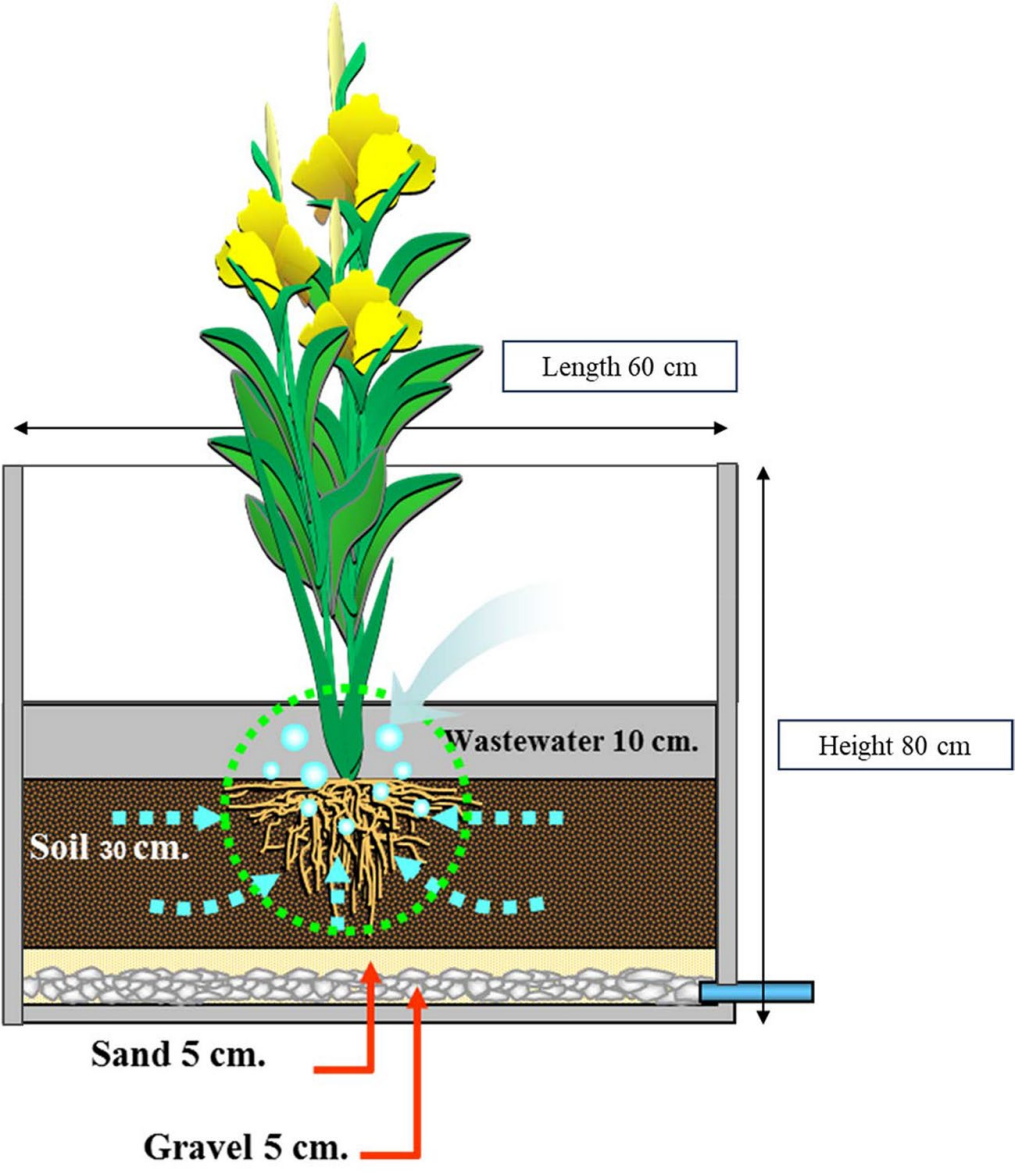
Schematic diagram showing an experimental pot with a vertical flow constructed wetland (VFCW) system growing a *Canna indica* or *Heliconia psittacorum* plant

The system comprised 50-cm-long concrete pots, each with an inner diameter of 100 cm. Each pot had a circular opening at the bottom where a PVC tube was attached to empty the contents into an effluent collector to control the water level in VFCW system. Each pot was paved with a 5-cm-thick gravel layer and gravel size diameter of 1.0-1.5cm, 5-cm-thick sand layer, and a 30-cm-thick soil layer, leaving free space up to a 10-cm height on the top for draining MW, where the influent of the MW was collected from the Phetchaburi municipality and transferred though the 18.5km HDPE pipeline as this process underwent mainly the anaerobic digestion process. Young plants (height: 30 cm) of *Canna indica and Heliconia psittacorum* were planted at the beginning of the experiment. In the preparation process, the plants rhizomes were nursing until new shoots of about 30 cm were formed. From these new shoots the sprouts were cultivated in the treatment plots. The initial characterization for heavy was not necessary as the plant shoots were assumed that there was no accumulation of heavy metals. MW was drained into each pot for 7-day cycle that included a 5-day flooding and 2-day drying. The experiment units were three pots each of *Canna indica* and *Heliconia psittacorum* with the planting density being 10 plants per square meter. Every week, the effluent in each pot was collected to determine the water quality, and the plant height was measured to determine its growth. The plants were harvested in the flowering stage for determining biomass production, nutrient uptake, and heavy metal accumulation, especially for lead and cadmium. The experiment was conducted from January to April of 2020 (Fig. 2).

### Water analyses

Each pot’s influent and effluent were examined every week to determine their characteristics, including the BOD, COD, TN, NH_4_-N, TP, lead, and cadmium, following the standard method for wastewater. The analysis methods for all the aforementioned parameters were formulated following APHA (1998), Marin and Ayele (2003), Maine et al. (2006), Hasan et al. (2007), Khan et al. (2009), and Penha-Lopes et al. (2012).

### Plant analyses

After 15 weeks, the plants in the VFCW units showed a zero-growth rate. They were harvested to determine the biomass and heavy metal accumulation in the aboveground (shoot) and underground (root) biomass growth, lead, and cadmium concentration. The biomass analysis involved cleaning the plant samples thoroughly, followed by air-drying them. Subsequently, the samples were separated into aboveground and underground portions, and their fresh weights were recorded at 1 kg each. The samples were then cut into smaller pieces and placed in Kraft paper envelopes (21 cm × 29.7 cm). These envelopes were oven-dried at 80 °C until a constant dry weight was achieved. Finally, the total biomass dry weight for each experimental unit was calculated (Huang et al. 2019). The analysis to determine the presence of cadmium and lead in plant samples was conducted by initially grinding the dried samples into a fine powder. Plant samples weighing 0.4 g were then digested using a mixture of concentrated nitric acid and perchloric acid in a 5:2 ratio, with a total volume of 10 mL. The digestion process continued until a clear solution was achieved. After cooling, the solution was filtered using Whatman No. 42 filter paper and adjusted to a final volume of 50 mL. Subsequently, the resulting solution was analyzed for cadmium and lead content using an Atomic Absorption Spectrophotometer, specifically the Perkin-Elmer Model 3300. (Bhowmik et al. 2012)

### Statistical analyses

The experiment conducted in this study was designed with a completely randomized block design. The effects of the following factors were analyzed: the plant species (C. *indica* and H. *psittacorum*) and wastewater pollutant removal efficiency among plants were also analyzed. The data were statistically analyzed by ANOVA, Student’s *t*-test, and Duncan’s multiple range test (DMRT). The least significant differences were calculated when the *p*-value was significant at the 0.05 level of significance.

## Results and discussion

### Characterization of influent wastewater and soil

The characteristics of the Phetchaburi MW, which drained into the VFCW experimental units with *Canna* and *Heliconia* plants, are listed in Table 1. The main pollutants in the MW were organic substances and nitrogen and phosphorus, originating from anthropogenic activities.

**Table 1:** Characteristics of Phetchaburi municipal wastewater in the vertical flow constructed wetland (VFCW) experimental units

Parameter	Unit	Influent	Standard*
pH	-	7.8 ± 0.3	5.5–9.0
BOD	mg/L	31.2 ± 13.9	< 20.0
TN	mg/L	22.7 ± 5.3	< 20.0
NH_4_^+^-N	mg/L	19.5 ± 5.8	-
TP	mg/L	4.0 ± 0.1	< 2.0

*Notifications of the Ministry of Natural Resource and Environment Re: the standard effluent discharge from domestic wastewater treatment system, B.E. 2553

The soil used for the experiment was mainly sandy clay loam. It consisted of 63% sand, 16% silt, and 21% clay and had neutral pH (7.0). Its organic matter content was very low (1.4%). The pollutant removal mechanism in wetland systems, specifically in the VFCW system, is related to the physical, biological, and chemical processes occurring between the soil and plant. These processes include filtration, sedimentation, microbial activity, plant uptake, and soil adsorption (Stefanakis et al. 2014), as the mechanism in explaining for the treatment process were that the canna roots structure provides high aerobic condition that supports the microbial process allowing for the removal of contaminants under chemical, physical and biological processes. These removal functions were classified under the phytoremediation process (Kulshreshtha et al. 2022). Studies from (Shah et al. 2023; Muduli et al. 2023) also suggested for a 59.9% and 73.4–90.6% removal efficiency of BOD under an onsite treatment of sewage and residential wastewater. While in the application of the usage of ornamental flowing plants, (Marín-Muñiz et al. 2020) suggested that a reduction of 30–90% of organic compounds, 30–70% of heavy metals and 99.99% of pathogenic bacteria was removed under the used of *Canna hybrids*.

### Plant growth and biomass production

**Fig. 3:**
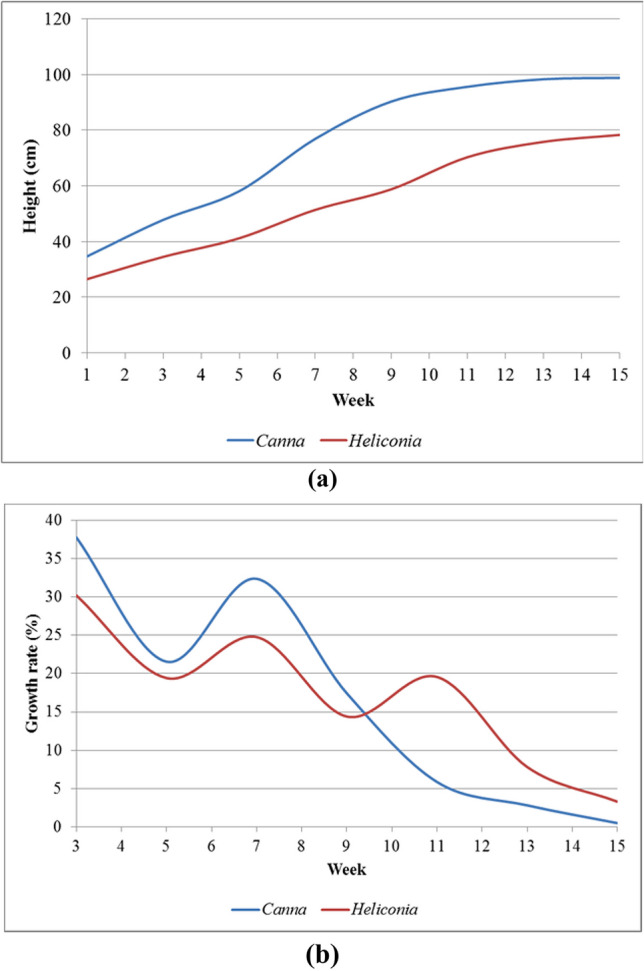
Growth of *C. indica* and *H. psittacorum* within 15 weeks in VFCW experimental units. (**a**) Height growth (**b**) Relative growth rate (RGR)

The Canna and Heliconia plants were used in the VFCW experimental units for MW treatment to tolerate the anaerobic condition under flooding. In addition, these ornamental plants can increase wetlands’ aesthetic and economic value. Both species grew well in the system, and although the *Canna* plants grew significantly taller than the *Heliconia* plants (*p* < 0.05), their relative growth rates were not statistically different. (Fig. 3).

Both species were harvested. The wet and dry weights of the aboveground and underground parts were measured. The measurements are listed in Table 2. Although the two species differed significantly in terms of their total biomass, the biomass production in aboveground and underground parts did not differ. Previous studies demonstrated that the biomass yields and relative growth rates of different species of emergent plants under flooding conditions are significantly different; for example, the biomass yield of *Canna* is greater than that of *Typha* or *Cyperus* (Phewnil et al. 2014). Further, higher water level or deeper burial inhibited, and more biomass was allocated to leaves at the 40 cm water level. Burial depth inhibited only plant growth in the absence of flooding (Pan et al. 2012). Hence, the biomass yield in this study was not affected by the water level because biomass allocation was affected by the water level. These findings highlight that plant adaptability, which depends on plant growth, community structure, nutrient removal rates, and potential for root zone aeration, is fundamental to ensure the maximum treatment results from the plants in a wastewater treatment system (Leto et al. 2013; Liang et al. 2011; Tanner 1996).

**Table 2:** Biomass production in the aboveground and underground parts of *Canna indica* and *Heliconia psittacorum* in the VFCW experimental units

Plant	Aboveground (g/plot)		Underground (g/plot)		Total biomass (g/plot)	
	Wet weight	Dry weight	Wet weight	Dry weight	Wet weight	Dry weight
*C. indica*	1.930^a^	170^a^	1.850^a^	140^a^	3.783^a^	277^a^
*H. psitacorum*	380^b^	40^b^	380^b^	40^b^	608^b^	85^b^

### Wastewater treatment efficiency of plants

Table 3 lists nutrient and pollutant removal rates. The BOD removal efficiency in both units exceeded 85%, and *Canna* showed significantly higher removal efficiency than *Heliconia*. The COD removal did not vary between the two plant species. The nutrient removal efficiency indicated that *Canna* removed more TN, NH_4_-N, and TP (*p* < 0.05). Konnerup et al. (2009) demonstrated that the COD mass removal rates of *Canna* and *Heliconia* vary between 42% and 83% depending on the loading rate of municipal wastewater. The present study used MW with high COD concentration and wastewater characteristics similar to those of the campus wastewater used by Konnerup et al. (2009). The results showed that COD removal was in the range of 75%, which is consistent with the previous findings of Calheiros et al. (2007) and Abou-Elela and Hellal (2012).

**Table 3:** Pollutant removal efficiencies of *Canna indica* and *Heliconia psitacorum*

Parameter	*Influent (mg/L)*	*Effluent (mg/L)*		Efficiency (%)		*p*-value
		*C. indica*	*H. psitacorum*	*C. indica*	*H. psitacorum*	
BOD	31.2 ± 13.9	2.5 ± 0.4	3.8 ± 0.5	90.5 ± 4.8	86.9 ± 7.3	0.012*
COD	49.7 ± 20.1	3.9 ± 0.6	5.7 ± 0.7	75.5 ± 7.9	75.3 ± 9.0	0.794*
TN	22.7 ± 5.3	12.6 ± 2.4	13.9 ± 3.8	44.3 ± 5.3	38.7 ± 2.7	0.002*
NH_4_^+^-N	19.5 ± 5.8	9.5 ± 3.0	10.1 ± 4.6	56.9 ± 13.4	50.0 ± 9.4	0.001*
TP	4.0 ± 0.1	1.7 ± 0.3	2.1 ± 0.2	56.7 ± 8.2	49.1 ± 7.3	0.001*

The removal of nitrates and BOD differed among the plant species because of the differences in chlorophyll fluorescence and photosynthetic characteristics, which led to different root lengths. The root biomass and structures of roots and rhizome influenced the removal of ammonia and total dissolved phosphorus and COD resulting from filtration by the root system; plants of the *Canna* and *Heliconia* species have fibril roots (Zhang et al. 2009; Li et al. 2013). The microorganisms in the root zone played an important role in determining the nutrient removal rate. The experiment units were mostly anaerobic d uring the 5-day periods of flooding, and nitrogen mineralization was the dominant microbial process during plant growth. Ammonium production and nitrogen assimilation comprised the main process under anaerobic conditions (i.e., the flooding period). Nitrification was the most important process under aerobic conditions (i.e., the drying period), which were expected to persist adjacent to plant roots because of the exudation of oxygen and carbon compounds to support microbial growth (Edwards et al. 2006). Ammonium uptake rate was significantly higher than nitrate uptake rate, which correlated to the growth rate because the ammonium significantly correlated with urease and protease activities. Plants and soil reduced nitrate but resulted in significantly greater nitrate reduction than that in the case of soil alone (Kong et al. 2009; Kearney and Zhu 2012). The nitrate transported in the plasma membrane of the root cells and nitrate reductase activity were induced by external nitrate (Zhang et al. 2007; Jampeetong et al. 2012). In addition, phosphorus mineralization processes dominated under anaerobic conditions. Phosphorus consumption by microbes was dominant in the aerobic part of the rhizosphere, and phosphorus removal significantly correlated with phosphatase activity (Edwards et al. 2006; Kong et al. 2009). Liu et al. (2012) reported that nutrient removal by soil in the presence of plant was significantly higher than that of unplanted soil. However, most ornamental plants adapted to the wastewater treatment systems well.

### Heavy metal accumulation in plants

Heavy-metal-contaminated MW in various facilities such as Bueng Makkasen in Bangkok contained manganese, iron, zinc, copper, lead, and cadmium (Chunkao et al. 2012). The dominant heavy metal contaminants in Phetchaburi MW were lead and cadmium. After harvesting, plants of both species were analyzed for lead and cadmium concentrations in both the aboveground (shoot) and underground (roots) parts, as specified in Table 4. The maximum lead and cadmium accumulations in the two plants did not differ between plant species; the exception was *Canna*. The lead concentrations differed significantly between the aboveground and underground parts, as indicated in Table 4. Generally, heavy metal accumulation in the underground part was greater than that in the aboveground part, and it was especially distributed in root tissues (Zhang et al. 2010). The factors that affect metal accumulation by wetland plants include metal concentration, pH, and nutrient status in the substrata (Deng et al. 2004). *Canna* and *Heliconia* absorbed less cadmium than lead because cadmium can accumulate in their tissues. They do not exclude it; cadmium is immobilized through cell-wall binding, and the thiol-rich peptides synthesized in the presence of cadmium may participate in cadmium-binding (Nyquist and Greger 2009). In addition, the selection and management of the cutting period, harvest period, frequency and time of harvest, and replanting period may notably affect the removal of metals (Guittonny-Philippe et al. 2014). In comparing the findings with other related studies, it has been suggested that the heavy metal (lead) reduction by *Phragmites australis* are that of 20 to 55% (Guzman et al. 2022). While in the use of *Canna indica* by subsurface flow of cadmium ions suggested that there was a removal percentage of 82% (Faisal et al. 2023). In the study on the performance of the vertical flow constructed wetland by *Canna* spp. varying the hydraulic retention time or 24–96 h, where it was suggested that longer HRT promotes higher chromium concentration. (Kumari and Dutta 2023)

**Table 4:** Lead and cadmium concentrations in the aboveground and underground parts of *C. indica* and *H. psittacorum* after harvesting

Parameter	Plant	Concentration (mg/kg)		*P*-value
		Aboveground	Underground	
Pb	*C. indica*	2.6 ± 0.4	3.2 ± 0.6	0.002*
	*H. psitacorum*	3.5 ± 1.1	3.5 ± 0.3	1.000
Cd	*C. indica*	0.4 ± 0.1	0.5 ± 0.1	0.288
	*H. psitacorum*	0.4 ± 0.1	0.5 ± 0.1	0.288

### Influence of *Canna* variety on wastewater treatment

**Fig. 4:**
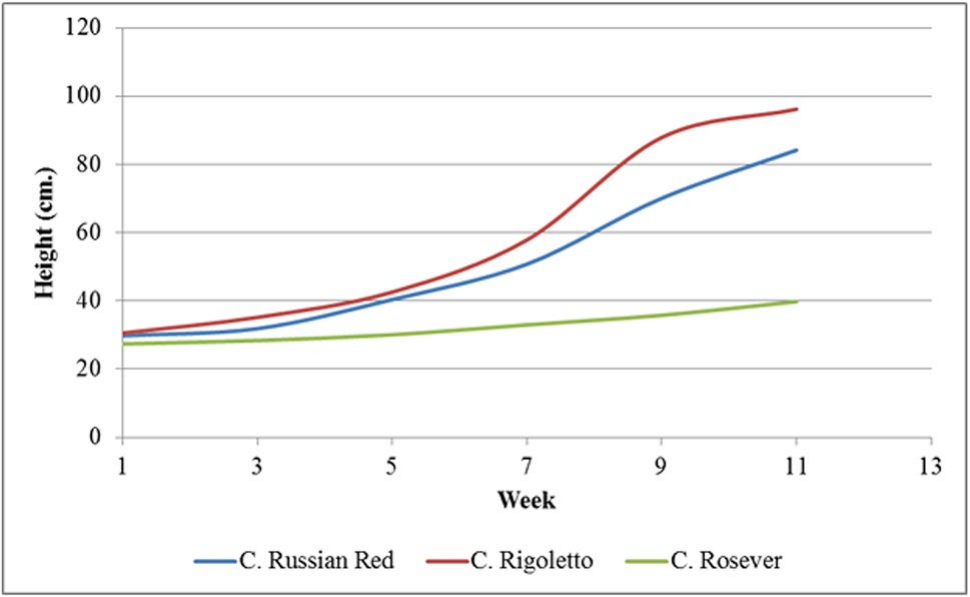
Height growths of *Canna* Russian Red, *Canna* Rigoletto, and *Canna* Rosever in the VFCW experimental units

**Fig. 5:**
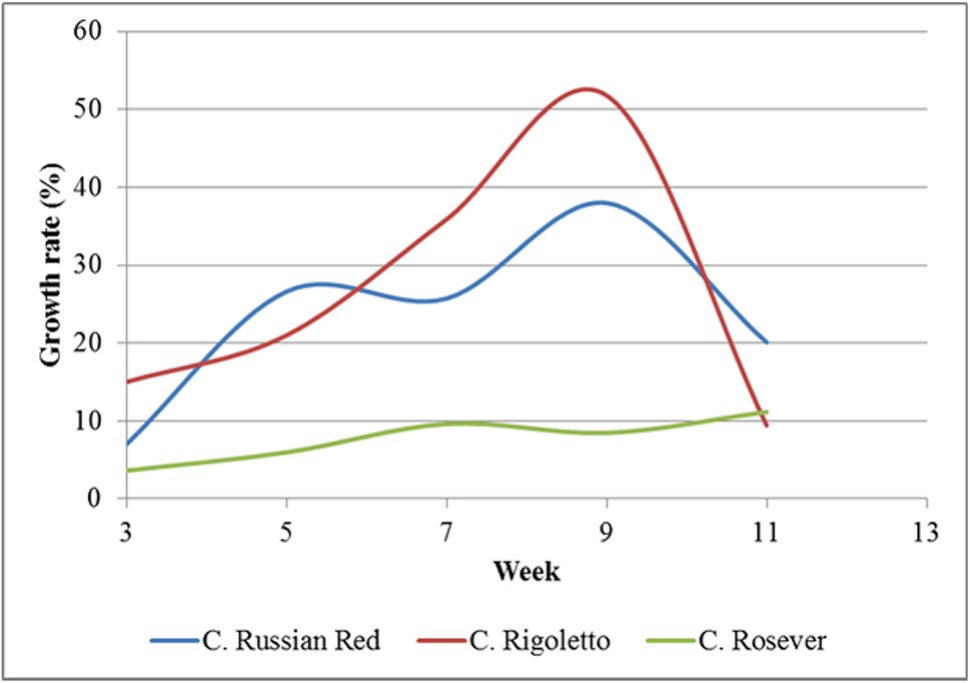
Relative growth rate of *Canna* Russian Red, *Canna* Rigoletto, and *Canna* Rosever in the VFCW experimental units

The results discussed in the previous section, *Canna indica* grew very well in the VFCW system with alternated 5-day flooding and 2-day drying. There are many varieties of *Canna* in Thailand. *Canna* Russian Red, *Canna* Rigoletto, and *Canna* Rosever were planted in VFCW experimental units with three replicates of each variety. The results demonstrated that the height growths of the species and the relative growth rates were significantly different (*p* < 0.05) because of the physiology of each plant, as shown in Figs. 3b and 4. The total biomass productions of *C.* Russian Red and *C.* Rosever were greater than those of *Canna* Rigoletto. The biomass yields in the aboveground parts of all varieties were significantly greater than those in the underground parts specified in Table 5. Similar findings from (Marín-Muñiz et al. 2023) suggested that there was also a significant difference in the growth characteristic in total biomass (root length and volume and height) between different *Canna hybrids* (Fig. 5).

**Table 5:** Biomass productions of *Canna* Russian Red, *Canna* Rigoletto, and *Canna* Rosever

Plant	Aboveground (kg/plot)		Underground (kg/plot)		Total biomass (kg/plot)	
	Wet weight	Dry weight	Wet weight	Dry weight	Wet weight	Dry weight
*C.* Russian Red	2.82^a^	0.55^a^	0.46^a^	0.11^a^	3.28^a^	0.65^a^
*C.* Rigoletto	3.87^b^	0.64^a^	0.52^a^	0.10^a^	4.40^b^	0.73^a^
*C.* Rosever	2.95^a^	0.45^a^	0.46^a^	0.09^a^	3.39^a^	0.54^a^

The results listed in Table 6 indicate that the nutrient removal efficiencies, including the BOD, TN, ammonia, and TP, for all plants exceed 95%, and there were no differences among the *Canna* varieties. Furthermore, the results demonstrate that nitrogen uptake (phosphorus uptake) by *C.* Rosever is greater than that by *Canna* Russian Red (*Canna* Rigoletto). With related findings from (Singh and Vaishya 2022) presented the application of a two-stage vertical flow hybrid-constructed wetland by of *Canna indica* in India removal efficiency of BOD, Nitrogen (NH_3_-N and NO_3_-N) and total phosphorus at be at 92.75%, 99.09%, 96.05%, and 88.83%, respectively. While furthering explain in the nitrogen removal phenomena (Wang et al. 2019) suggested that the effects of root exudates by macrophytes positively correlates with nitrate-reducing bacteria (denitrification) in which nitrites are converted into ammonia under anoxic condition, however, it was during the 2-day dry period promoting an aerobic condition in the treatment plots supporting the nitrification process (Hernández et al. 2018). In the phosphorus removal, it has been suggested that the substrates were in the condition of the main pathways of phosphorus that can be uptake by *Canna* hybrid through assimilation have suggested for a 45% removal efficacy. (Quan et al. 2016) The nitrogen uptake was greater than the phosphorus uptake, and greater amounts of nitrogen and phosphorus were accumulated in the aboveground parts than in the underground parts, as presented in Table 7.

**Table 6:** Pollutant removal efficiencies of *Canna* Russian Red, *Canna* Rigoletto, and *Canna* Rosever

Parameter	*C.* Russian Red (%)	*C.* Rigoletto (%)	*C.* Rosever (%)
BOD	96.2 ± 1.9^a^	94.5 ± 5.8^a^	95.7 ± 4.1^a^
TN	95.2 ± 2.1^a^	96.6 ± 1.0^a^	96.2 ± 1.9^a^
NH_4_^+^-N	98.1 ± 1.0^a^	98.1 ± 0.8^a^	97.9 ± 0.9^a^
TP	99.2 ± 0.2^a^	99.0 ± 0.2^a^	99.2 ± 0.2^a^

**Table 7:** Nutrient uptakes of *Canna* Russian Red, *Canna* Rigoletto, and *Canna* Rosever

Plant	Aboveground (g/plot)		Underground (g/plot)		Total biomass (g/plot)	
	N	P	N	P	N	P
*C.* Russian Red	45.71^b^	5.41^b^	0.46^a^	0.07^b^	46.17^b^	5.48^b^
*C.* Rigoletto	52.44^c^	5.96^b^	0.43^a^	0.06^b^	52.87^c^	6.02^b^
*C.* Rosever	29.45^a^	2.82^a^	0.29^a^	0.04^a^	29.74^a^	2.86^a^

## Conclusions

Ornamental plants such as *Canna* and *Heliconia* can be used in VFCWs under alternating 5-day flooding and 2-day drying conditions. They grow well in such systems and can develop a deep and dense root system. They can be applied for pollutant removal to remediate the BOD, COD, TN, ammonium, and TP in the presence of multiple pollutants, as is commonly found in municipal wastewater. The heavy metal accumulation in the two plants did not differ significantly between plant species, though lead and cadmium accumulation in *Heliconia* were slightly greater than their accumulation in *Canna*. The exception was that in the case of *Canna*, the lead concentrations of the aboveground and underground parts differed significantly. *Canna* is the preferred species for VFCW with an alternated flooding and drying system because it exhibits more vigorous growth and high pollutant removal efficiency. Its flowers increase the aesthetic value of the wastewater treatment site.

## Data Availability

The datasets used and analyzed during the current study are available from the corresponding author on reasonable request
